# Improved YOLOv5-based for small traffic sign detection under complex weather

**DOI:** 10.1038/s41598-023-42753-3

**Published:** 2023-09-27

**Authors:** Shenming Qu, Xinyu Yang, Huafei Zhou, Yuan Xie

**Affiliations:** School of Software, Henan University, Kaifeng, 475004 Henan China

**Keywords:** Electrical and electronic engineering, Energy science and technology

## Abstract

Traffic sign detection is a challenging task for unmanned driving systems. In the traffic sign detection process, the object size and weather conditions vary widely, which will have a certain impact on the detection accuracy. In order to solve the problem of balanced detecting precision of traffic sign recognition model in different weather conditions, and it is difficult to detect occluded objects and small objects, this paper proposes a small object detection algorithm based on improved YOLOv5s in complex weather. First, we add the coordinate attention(CA) mechanism in the backbone, a light-weight yet effective module, embedding the location information of traffic signs into the channel attention to improve the feature extraction ability of the network. Second, we exploit effectively fine-grained features about small traffic signs from the shallower layers by adding one prediction head to YOLOv5s. Finally, we use Alpha-IoU to improve the original positioning loss CIoU, improving the accuracy of bbox regression. Applying this model to the recently proposed CCTSDB 2021 dataset, for small objects, the precision is 88.1%, and the recall rate is 79.8%, compared with the original YOLOv5s model, it is improved by 12.5% and 23.9% respectively, and small traffic signs can be effectively detected under different weather conditions, with low miss rate and high detection accuracy. The source code will be made publicly available at https://github.com/yang-0706/ImprovedYOLOv5s.

## Introduction

With the rapid development of computer science and artificial intelligence, object detection algorithms have been widely used in areas such as unmanned driving and video indexing. It is very important for unmanned vehicles to accurately detect and identify vehicles, traffic signs, and signal lights in road scenes during driving. Traffic signs on urban roads contain abundant navigation information, which can indicate and warn drivers’ driving behavior. It is very important to detect and recognize the traffic sign correctly, and it is an important component of the intelligent driving system^1^.

Three major problems need to be overcome in traffic sign detection tasks. Firstly, traffic sign detection is a small object detection method^2^, the small object has a small number of pixels, carries limited information, and makes a lot of noise. Therefore, small objects pose a significant challenge to feature extraction. The second problem is that the weather is complicated, and it is easy to miss the object. The last problem is that the kinds of traffic signs are not balanced, and in the actual detection process, the probability of the occurrence of different kinds of traffic signs is different.

Before deep learning is widely used, color, shape, and machine learning-based methods are usually used for detection and classification in previous traffic sign recognition tasks. Fleyeh et al.^3^ uses color segmentation in traffic sign detection task, which is on the basis of AdaBoost binary classifier and cyclic Hough transform. This method has high accuracy and good robustness. Piccioli et al.^4^ perform edge detection on true roadway traffic sign images and then located them in the images according to the shape characteristic of the traffic sign. In the German traffic sign competition, Wang et al.^5^ use HOG and SVM to detect traffic signs and achieved good results.

With the development of computer technology, the security of deep learning has also been improved to a certain extent. A federated deep learning method^6^ has been proposed to make the deep learning method more reliable. Some new approachs have been proposed to detect traffic signs, which is based on a convolutional neural network (CNN)^7^. This method sends traffic sign data samples and label files to the input of the network, and extracts image features by adjusting the combination of network layers and the size of the convolution kernel. The continuous evolution of the network parameters enables the algorithm to learn the transformation relationship between different traffic signs and avoids some errors of artificial feature extraction.

Compared with the traditional traffic detection, the method based on deep learning improves the detection accuracy and speeds up the detection speed. However, due to the variable size of traffic signs, there is a significant gap between the detection of small objects and normal objects, and most algorithms are still not ideal for the detection of small traffic signs. At the same time, complex weather conditions also lead to a high rate of missed detection and false detection in most algorithms under bad weather conditions.

In response to the above issues, this paper adopts the single-stage network model YOLOv5s with excellent accuracy and speed as the benchmark model and integrates the coordinate attention(CA)^8^ mechanism. The CA filters out redundant feature information, keeps the key features, and improves detection accuracy. In addition, we also added a small object detection layer to make our network pay more attention to the detection of small-scale traffic signs and improve the overall accuracy of the model.

The main contributions of our work are summarized as follows:

- For complex weather conditions, the CA is added to the backbone network. By inserting location information into channel attention, the network can obtain more extensive regional information and achieve accurate object detection.
- For small traffic signs, we propose adding a small object detection layer to the existing network to reduce missed detection of small objects and improve the detection accuracy of small objects.
- Unlike the existing YOLOv5s network, the current version is improved to reduce the impact of scale variability. Meanwhile, it can be deployed on the mobile terminal of the vehicle to detect and recognize traffic signs in real-time.

## Related work

Object detection is a key task in Computer Vision (CV). It is also the basis for many complex tasks, such as object tracking and abnormal behavior detection. Recently, the CNN has achieved remarkable results in object detection due to its outstanding performance. Many researchers have begun to apply these methods to traffic sign data. Next, we briefly discuss the object detection algorithms based on CNNs and their applications in the field of traffic sign detection.

### CNN-based detection

There are two main types of traffic sign detection methods: single-stage detection and two-stage detection, two-stage detection method first determines the region of interest by retrieving the approximate location of the object, and then uses a feature extraction network to determine the coordinates and specific categories of the object. Single-stage detection method can achieve the whole process from images to the classification results within a network.

#### Two-stage detection

The training process of the two-stage series of algorithms is divided into two parts: generating candidate regions (region proposals), then classifying and regressing these candidate regions, and training the RPN network for object region detection. The accuracy of this method is better than that of single-stage method, The typical representative is a series of algorithms represented by R-CNN^9^, which first adopts the selective search^10^to obtain about 2k region proposals, then adopts the CNN to extract the features of the region suggestions, and finally determines the category of objects through multiple SVMs^11^,the linear regression is used to finetune the boundary. The SPP-Net^12^ extracts features from the entire image through a single convolution, avoiding the problem of a large amount of redundant computation. However, like RCNN, the SPP-Net also uses almost the same multi-level pipeline, which still requires extra storage space costs. Fast R-CNN^13^ simplifies the SPP layer to an ROI pooling layer based on SPP-Net. Only the input image is fed back to the convolution layer, and the fixed-length feature vector is extracted by ROI pooling, but there is a issue with Fast R-CNN that the computation is too much.To address this issue, Ren et al. proposed a faster R-CNN^14^. The algorithm has been further improved by replacing selective search with a regional recommendation network (RPN), which shares its convolutional layer with classification and regression networks. Through this method, they achieve end-to-end computing for target detection, greatly improving detection efficiency through shared convolution layers. In general, the two-stage algorithm has high accuracy but low efficiency.

#### Single-stage detection

The single-stage algorithm transforms the frame position problem into a regression problem. The category and position information are given through the backbone network, and the RPN is not used, so the speed is more advantageous, but due to the uneven proportion of positive and negative candidate boxes, the accuracy will be lost compared to the two-stage algorithm. Single-stage algorithms mainly include Over Feat, YOLO (You Only Look Once) series, and SSD (Single Shot MultiBox Detector) series, among which two typical algorithms are the SSD series and YOLO series.

The Overfeat^15^ model is released in 2013. An ensemble framework for classification, localization, and detection using a convolutional neural network is proposed. At the same time, a new learning method is introduced, that is, localization is performed by predicting the object boundary, and the frame is accumulated to increase the detection confidence. In 2016, Redmon et al. proposes the YOLO algorithm^16^, which innovatively transforms the detection issue into a regression issue, and uses the CNNs to directly determine the object category and predict the boundary, opening a new era of the single-stage algorithm for object detection. YOLO series algorithms meet the real-time performance of object detection, but make certain sacrifices in detection accuracy, especially for small objects with dense distribution, YOLOv1 is more prone to missed detection. In recent years, the Redmon team has continuously improved the YOLO algorithm and updated it to YOLOv3^17,18^. In 2020, Bochkovskiy and others improves and releases YOLOv4^19^ on this basis, and the subsequent YOLOv5^20^ and YOLO X version changes made the YOLO series of algorithms more suitable for engineering practice, and their accuracy and real-time performance are improved, but there are still problems such as low object localization accuracy and poor recall rate need to be solved. In 2016, Liu et al.^21^ proposes the SSD algorithm, which solves the problems of low positioning accuracy and difficulty in detecting small objects that existed in the YOLO algorithm at the same time. By combining good points of the faster R-CNN algorithm and the YOLO algorithm, SSD fully mines the feature information of the convolutional layer by using the pyramid structure of the feature. The algorithm can guarantee the speed at the same time, and can overcome the disadvantages of YOLO in some degree. But the algorithm is difficult to detect small objects and has the disadvantage of inaccurate positioning. In 2017, Fu et al.^22^ further optimizes the SSD algorithm and proposes the DSSD(deconvolutional single shot detector) algorithm, which uses deconvolution to replace the traditional bilinear interpolation upsampling in the fusion module in SSD, and combines high-level semantics with high-level semantics. The low-level feature information is fully fused, which further improves the detection accuracy of small objects. But as the network becomes more complex, the speed of the algorithm is reduced.

At present, the above two methods are commonly used for traffic sign detection. Yang et al.^23^ designed a visual multi-scale attention module for traffic sign detection, which integrates multi-scale feature maps with channel weights and spatial masks. Zhang et al.^24^ designed a bottom-up enhancement path to enhance the feature pyramid, thereby effectively utilizing fine-grained features at the bottom to achieve precise positioning of traffic signs. But most CNN-based detection methods are designed for normal size objects. When detecting traffic signs, directly selecting these methods to detect traffic signs in an image can cause problems such as false detection and missing detection.

### Visual attention model

The attention mechanism is to obtain the difference in the importance of each feature map by certain means, put more computing resources of the neural network into more important tasks, and use the task results to reversely guide the weight update of the feature map, to complete the corresponding tasks efficiently and quickly. The attention mechanism is originally used as part of the encoder-decoder framework in RNN(Recurrent Neural Network) in 2014 to encode long input sentences and has been widely used in RNNs since then. With the wide application of deep learning, some scholars propose to introduce an attention mechanism in CNN and apply it to the feature map, trying to obtain the available attention information in the feature map to achieve better task effects. At present, the attention mechanism of CV is divided into channel attention mechanism, spatial attention mechanism, and mixture attention mechanism.

In 2016, Momenta^25^ proposes a squeeze-and-excitation(SE) block for learning the channel relationship of feature maps, which makes it win the 2017 ImageNet with an absolute advantage. In 2019, ^26^ is an enhanced version of SENet which can adaptively adjust different sizes of the receptive field. In 2018, Woo et al.^27^ proposes a kind of mixture attention model called convolutional block attention(CBAM). It combines the spatial attention module and channel attention module serially. Similar to CBAM, the bottleneck attention module (BAM)^28^ also has a spatial attention module and a channel attention module. The only difference between them is that BAM connects its two attention modules in parallel. The no-local series^29,30^ draws on the idea of non-local mean denoising in traditional CV. In^31^, an a spatial-spectral residual attention network (SSRAN) is proposed to simultaneously explore the spatial and spectral information of MSI for reconstructing the HSI. Zheng et al.^32^ proposes a rectified spatial attention (RSpaA) module that replaces the original convolution to extract rotationin-variant spectral-spatial features from HSI patches and enhance network performance. This points to the importance of the attention mechanism, a lesson echoed in our results. In 2021, Hou et al.^8^ proposed embedding position information into channel attention, decomposing channel attention into two one-dimensional feature encoding processes, and aggregating features along two spatial directions. Accurate location information can be preserved while capturing remote dependencies.

## The detection network for small traffic sign

The YOLOv5s network is mainly divided into the following four modules: Input , Backbone, Neck, and Prediction head. The backbone uses a cross stage partial network(CSP), the neck is composed of FPN+PAN^33^, and the prediction head selects three yolo heads, each of which matches three anchor. We have improved the backbone, prediction heads, and loss function sections of YOLOv5s to make it more suitable for small traffic sign detection.

### The network structure

**Figure 1:**
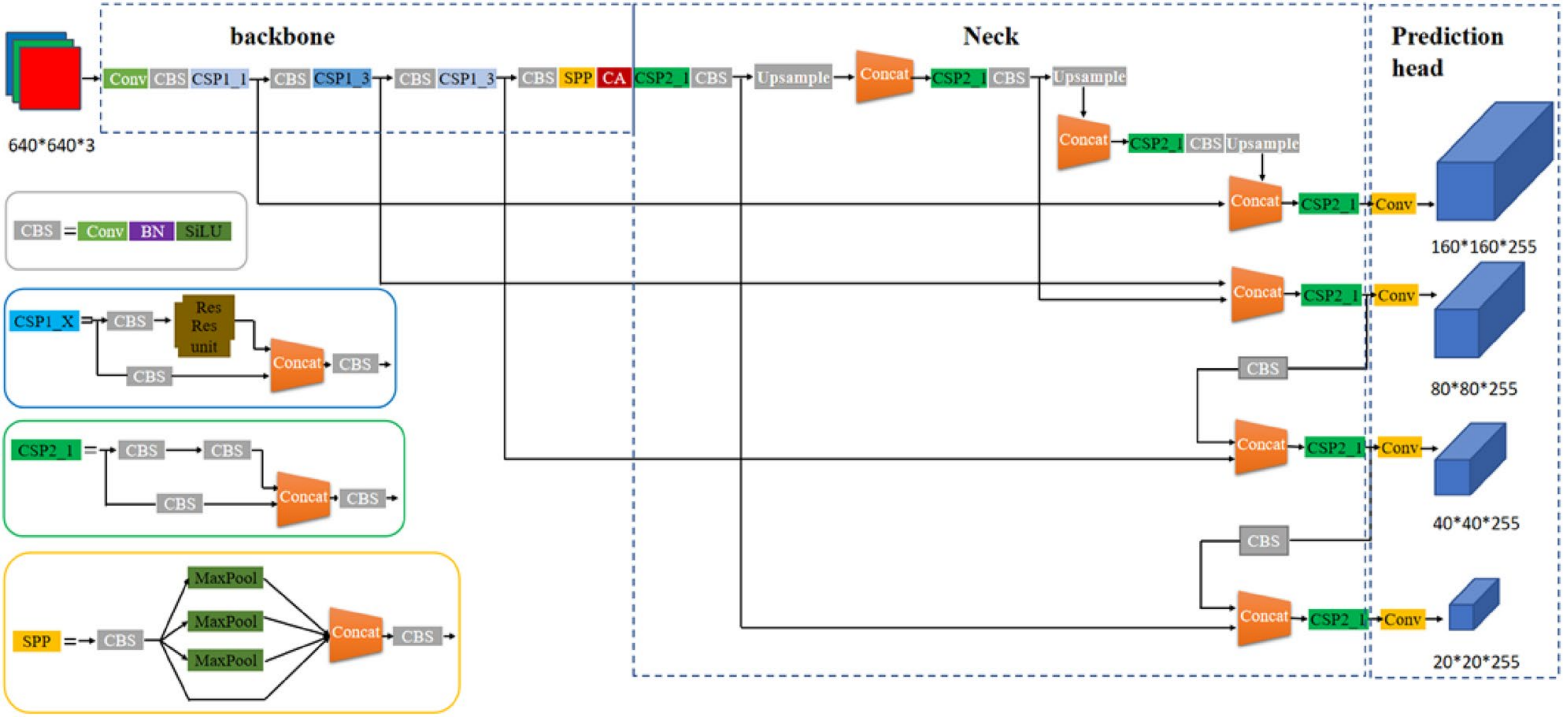
The architecture of the improved YOLOv5s network.

Here we describe the improvement of the network from the YOLOv5s in detail, as illustrated in Fig. 1.

In the backbone of the improved network, we have made some small changes to CSP that are more suitable for extracting features from small objects. On the original YOLOv5s, the input image needs to go through a Focus module, the Focus module does not lose information in the process of downsampling, which increases the batch size of the network. However, in a shallow network, retaining the above information has no obvious gain in network performance, more importantly, it is to reduce the floating point number and improve the running speed. So replace it with a large convolution kernel Conv(k $=$ 6, s $=$ 2, p $=$ 2) to further compress the module size and obtain better performance. At the same time, the CA is introduced at the end of the backbone network, embedded the location information of traffic signs into channel attention, enabling the improved network to obtain information in a larger area, effectively improving network performance.

For small traffic signs, we propose a small object detection layer and define a cross layer connection to implement this content, corresponding to the neck and prediction head parts in Fig. 1. In the prediction heads of improved network, we add a yolo-head to the original yolo v5s, which is used to detect small size traffic signs. A yolo-head’s tensor is $\hbox {M} \times \hbox {M} \times [3 \times (\hbox {N}+4+1)]$, which is used for N-type objects, 4 enclosure box offsets, and 1 objectness prediction. In our application, the categories of traffic signsn is equal to 3, so a yolo-head’s tensor is equal to 24. The increased yolo head which enable the network get more small object characteristics, achieve more intensive anchor sampling to effectively improve the detection accuracy of small objects.

### Coordinate attention module

The attention mechanism comes from people’s processing of image data. Through the observation of global information of the image, humans can use attention to lock the candidate region of focus, automatically shield some backgrounds and redundant information, and can quickly lock the focus^34^. The attention mechanism is an effective way to improve the ability of extracting the feature of neural networks.

**Figure 2:**
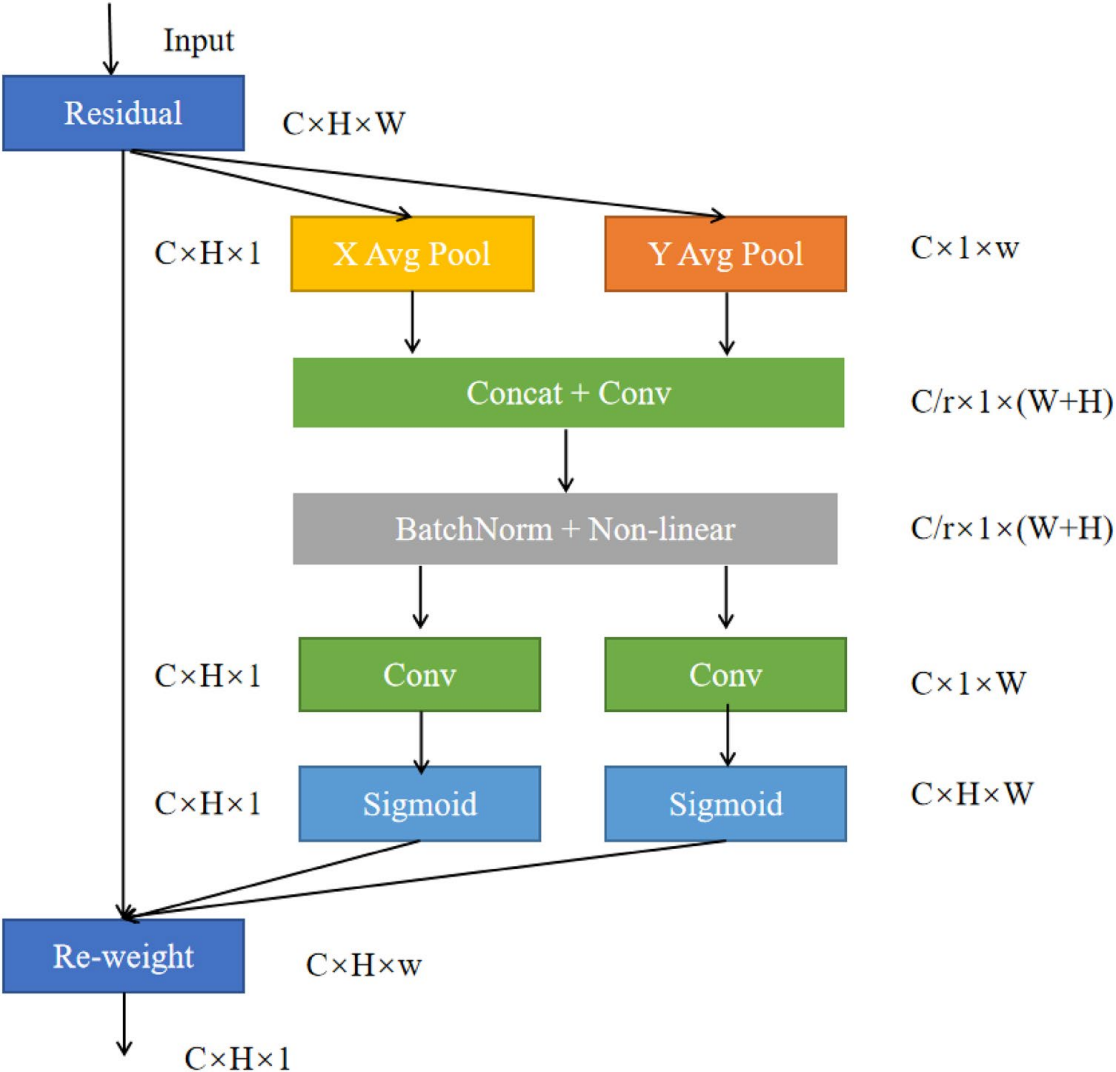
The structure of coordinate attention.

Because of the special position information of traffic signs, we introduce CA attention, and CA is a new kind of effective attention mechanism, which is to embed the position information into the channel attention. CA can not only capture the information of the channel but also capture the direction and location information, which can make the model more accurate in positioning and identifying important information. The CA module structure is shown in Fig. 2. First of all, the location information is embedded in the channel attention, so that the lightweight network can obtain information in the larger area, and reduce the parameters of the attention module while avoiding excessive calculation overhead. To avoid the loss of location information caused by the two-dimensional global pooling, the two-dimensional global pooling is decomposed into two parallel one-dimensional feature codes, which are converged along the horizontal and vertical directions, and the space coordinate information is integrated efficiently.

### The small object detection layer

To extract more shallow objects to improve the detection effect of small traffic signs, we have added a detection layer for small objects in the network. To focus on the detection of specific feature maps, we use a cross-layer connection in the backbone and neck, we continue to upsample the feature map at the last layer of the neck, so that the feature map continues to expand, and fuse the feature map obtained in this step with the first feature map obtained by the backbone network, to obtain a larger feature map for small object detection. In the prediction head part of the network, we add a yolo-head for small objects, corresponding to the first prediction head in Fig. 1. We use four yolo-heads to predict the bounding boxes and categories of traffic signs of four different scales, which can expand the size range of the detected objects.

### Loss function

In YOLOv5s, the loss function consists of three parts, which are confidence loss, classification loss, and positioning loss.

The confidence loss uses a cross-entropy loss function, as shown in Eq. (1):

1 $$\begin{aligned} {loss}_{conf}={\lambda _noobj}\sum _{i=0}^{S\times S}\sum _{j=0}^{B}{I_{i,j}^{noobj}\left( {Conf}_i-{\widehat{Conf}}_i\right) ^2}+\lambda _{obj}\sum _{i=0}^{S\times S}\sum _{j=0}^{B}{I_{i,j}^{obj}{\left( {Conf}_i-{\widehat{Conf}}_i\right) }^2} \end{aligned}$$

Among them, obj and noobj respectively indicate whether the object is required for detecting the detection layer, S$\times$S is the number of grids of the detection layer, B is the number of anchor boxes set artificially for each grid, ${Conf}_i$ is the confidence prediction, while $\widehat{Conf}_i$ is its label. $\lambda _noobj$ and $\lambda _obj$ are the weighted coefficients to balance the loss function. When there is no object to be detected in the j-th bounding box of the i-th grid, $I_{i,j}^{noobj}$ is equal to 1, if it exists, it is equal to 0, and $I_{i,j}^{obj}$ is exactly the same as the opposite.

The classification loss function is as shown in Eq. (2):

2 $$\begin{aligned} {loss}_{cla}={\lambda _{class}}\sum _{i=0}^{S\times S}\sum _{j=0}^{B}{I_{i,j}^{obj}\sum _{c\in classes}{p_{i,j}(c)\log ({\hat{p}}_{i,j}(c))}} \end{aligned}$$

Among them, C is the number of data sets, that is, 3, $\hat{p}_{i,j}(c))$ is the real probability of one of the types, $p_{i,j}(c)$ is a certain type of prediction probability, and only calculates the classification loss of the object that contains the required detection. $\lambda _{class}$ is a weighted coefficient to balance the loss function.

The original positioning loss uses CIoU^35^. In the traffic sign recognition task, there will be a large deviation between the predicted box and the true box. To solve the problem, we use Alpha-IoU^36^ to improve the CIoU. By adjusting ($\alpha>$ 1), the loss and gradient of high IoU objects are added, thereby improving the accuracy of bbox regression and realizing more accurate loss calculation between the predicted box and the true box in the traffic sign recognition task. First, we introduce the IoU, which is used to measure the similarity between the object detection algorithm and the actual bounding box. A threshold is set in advance, if IoU is greater than the threshold, the bounding box is determined to be predicted to be more accurate, otherwise, the prediction is determined to be wrong. The calculation formula of IoU is as shown in Eq. (3):

3 $$\begin{aligned} IoU=\left| \frac{B\cap B^{gt}}{B\cup B^{gt}}\right| \end{aligned}$$

Among them, B and $B^{gt}$ represent the prediction box and the real box, respectively. When using IoU as a performance measurement, if the two boundary boxes are completely overlapped, the value is 1. If the two objects do not overlap, the IoU and gradient are 0, which cannot be optimized; the improved loss function is shown in Eq. (4):

4 $$\begin{aligned} Alpha-IoU=1-{IoU}^\alpha +\frac{\rho ^{2\alpha }\left( b,b^{gt}\right) }{c^{2\alpha }}+{(\beta v)}^\alpha \end{aligned}$$

$\alpha$ is an additional Power regular item and takes 3 in this article.

The final positioning loss function is shown in Eq. (5):

5 $$\begin{aligned} {loss}_{loc}=\lambda \sum _{i=0}^{S\times S}\sum _{j=0}^{B}{I_{ij}^{obj}\left( 1-\textrm{AlphaIoU}_{ij}\right) } \end{aligned}$$

## Experiments

In this section, we comprehensively evaluate the improved YOLOv5s model through the CCTSDB 2021^37^ dataset. The CCTSDB 2021 randomly collected over 1000 car video tapes and effectively expanded the dataset by manually saving key frames with traffic signs. On the basis of CCTSDB 2017^2^, CCTSDB 2021 adds 5268 new traffic scene images, of which 3,268 training set images and 2,000 test set images. This dataset replaces some simple samples from the original standard sample set while expanding the sample size, making the network more robust. CCTSDB 2021 incorporates images from different scenarios to maximize the realistic driving environment.

**Figure 3:**
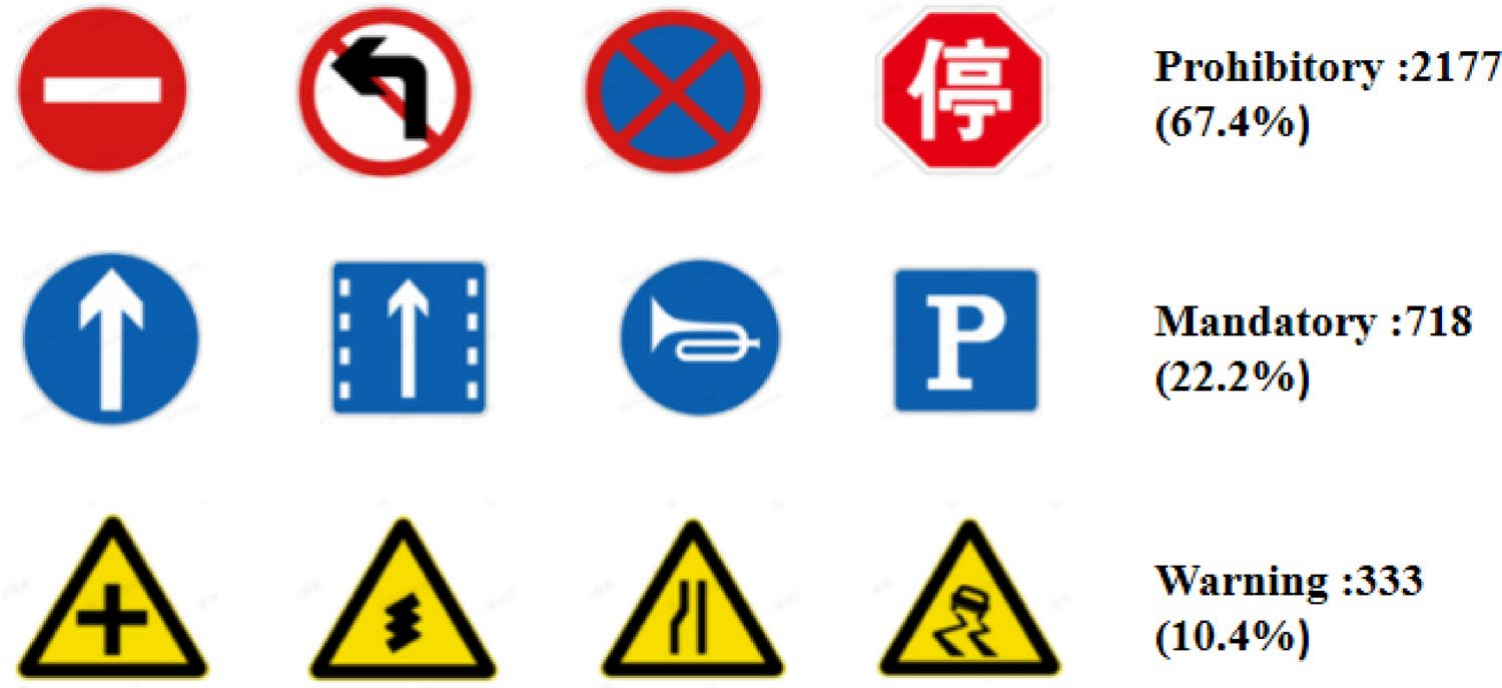
The CCTSDB 2021 Division of traffic signs and its proportion.

CCTSDB 2021 divides the samples in the test set in more detail according to three dimensions: category meanings (three types), sign sizes (five types), and weather con-ditions (six types), as detailed below. According to the definition of common road traffic signs, the signs appearing in the dataset are divided into three categories: prohibitory signs, mandatory signs, and warning signs. The graphical representation and proportion are shown in Fig. 3. According to the size of traffic signs, CCTSDB 2021 divides them into five categories, namely XS (access small), S (small), M (medium), L (large), and XL (extra-large). In the 1500 test set images of CCTSDB 2021, all sample images are classified into six categories based on weather illumination, namely foggy, snow, rain, night, sunny, and cloud.

### Experimental details

Considering the fixed size of input demanded by the YOLOv5s network, we resize the images to uniform dimensions of 640$\times$640. The training and validation datasets include 16,356 images and the test dataset includes 1500 images. In the process of training, the initial value of the learning rate was 0.001, with a momentum size of 0.98, and a weight decay parameter of 0.001. The epochs and the batch size are set to 100 and 32, respectively. The training and testing codes of all models run in the windows environment of CUDA 11.2, and the framework adopted for the experiments is PyTorch. The processor model is AMD CPU Ryzen 7 5800H, the graphics card model is GeForce RTX 3050, the graphics memory size is 8G, and the memory size is 16G.

**Figure 4:**
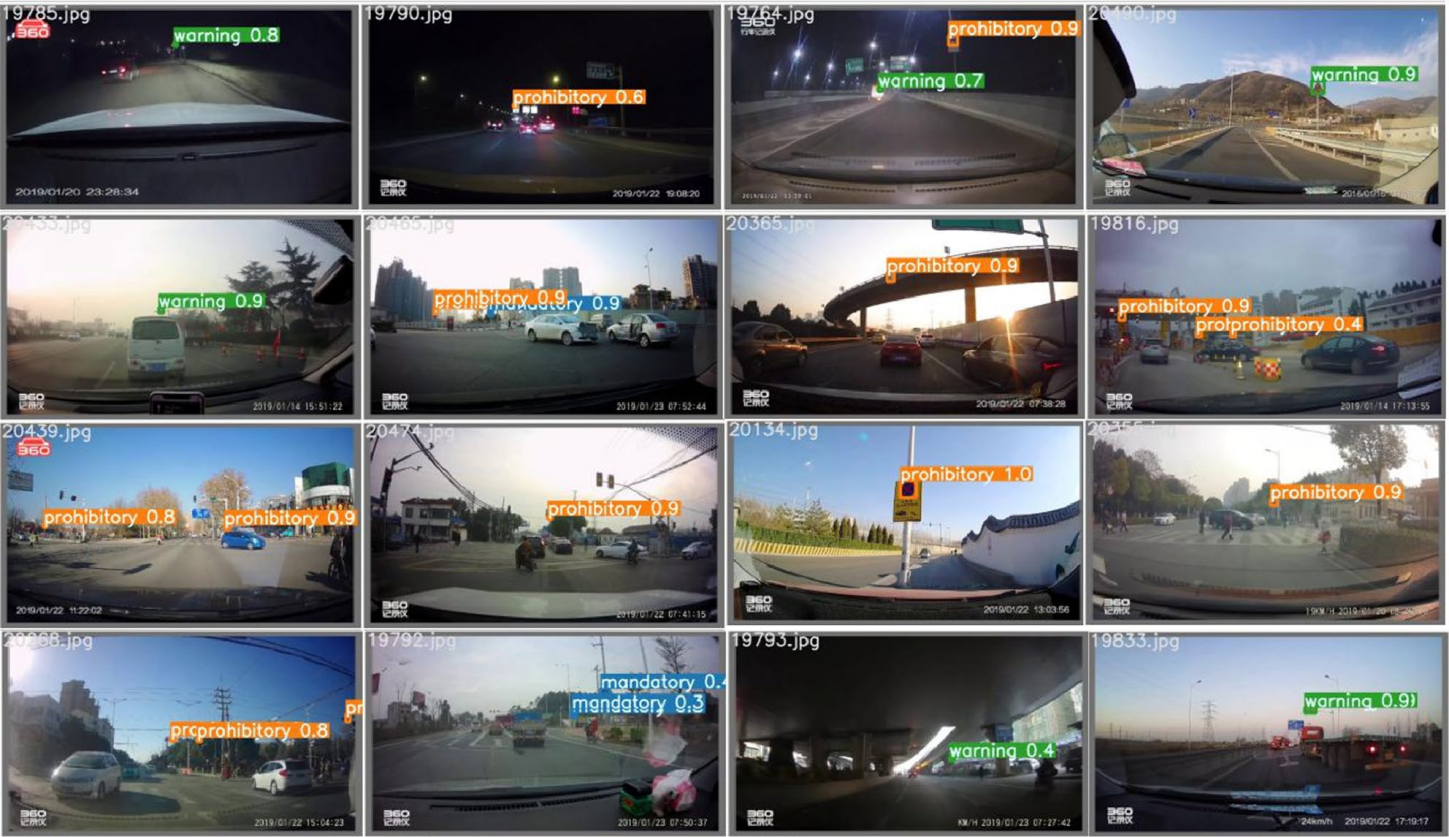
Some experiment results on the CCTSDB 2021.

**Figure 5:**
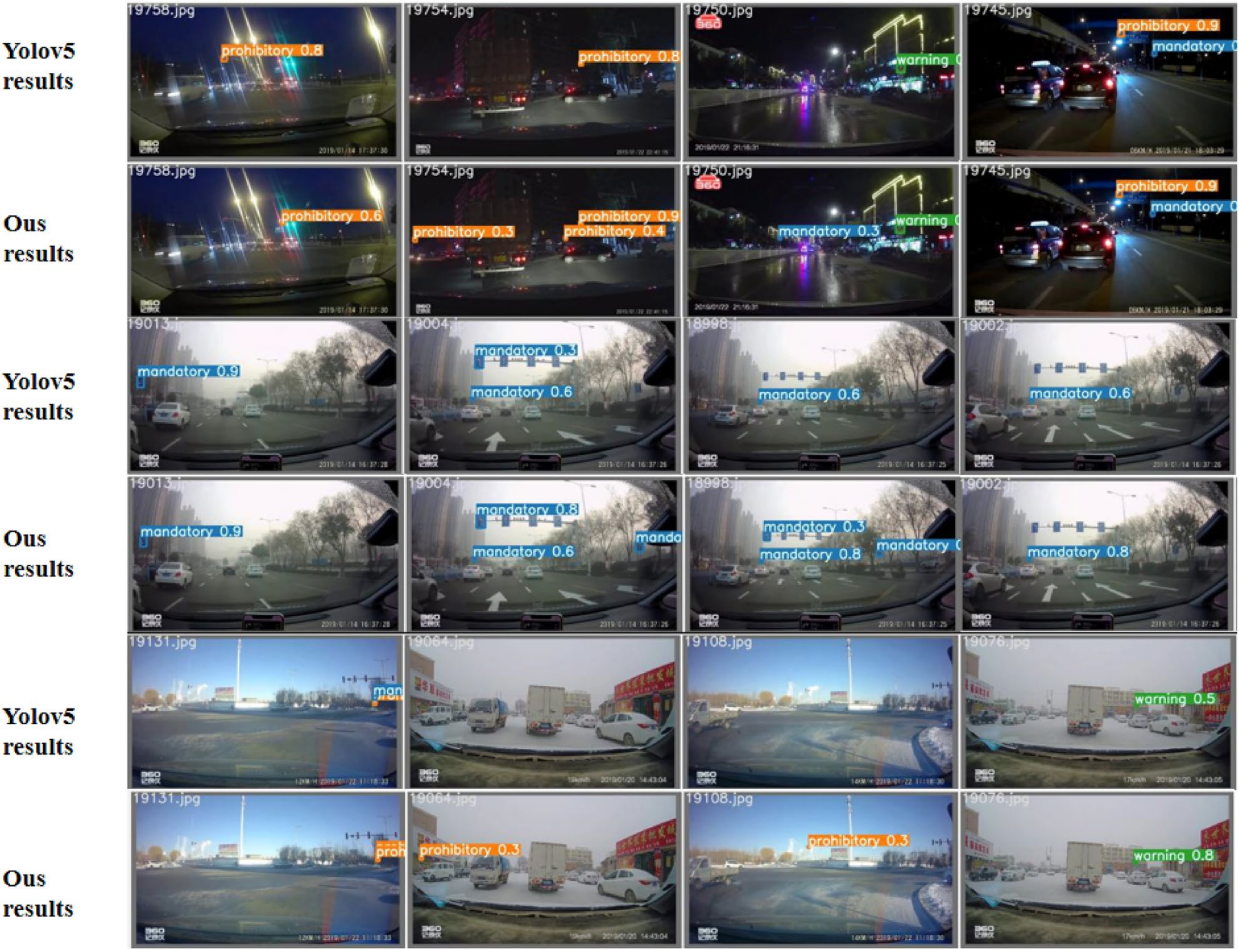
Results for some complex weather cases in the CCTSDB 2021 dataset.

Regarding the Figs. 4 and 5, we used Python to draw them. The sofware version is Python 3.7.6. The website link is https://www.python.org/downloads/release/python-376/.

### Experimental results

To demonstrate the advantages of the proposed method in traffic sign detection, we evaluated our method on the CCTSDB 2021 and compared it with the original YOLOv5^20^, Faster R-CNN^14^, SSD^21^, YOLOv3^18^, RetinaNet^38^, Dynamic R-CNN^39^, PSG-Yolov5^40^,and YOLOv4^19^. We evaluated performance using six common metrics including precision(P)^33^, recall rate(R)^33^, miss rate(MR)^33^, mean average precision(mAP)^41^, F1^41^, and frames per second(FPS), the metrics can be calculated according to the formula 6:

6 $$\begin{aligned} {} & precision=\frac{TP}{TP+FP}\\&recall=\frac{TP}{TP+FN}\\ & miss=1-\frac{TP}{TP+FN}\\ & mean\ average\ precision=\frac{1}{classes}\sum _{i=1}^{classes}\int _{0}^{1}{P(R)dR}\\ & F_1=\frac{2PR}{P+R}\\ & frames\ per\ second=\frac{N}{\sum _{j}^{N}T_j} \end{aligned} $$

Among them, TP represents the number of positive classes judged as positive, FP represents the number of negative classes judged as positive, and FN represents the number of positive classes judged as negative. N represents the number of processed images, and $T_j$ represents the time taken to process the jth image.

The specific results are shown in Table 1. We can see that our approach has reached 82.8% mAP on CCTSDB 2021 data set, which is higher than other models and an improvement of 6.5% compared to the original YOLOV5, and corresponding improvements in other indicators. Though the FPS value is lower than YOLOv5s, it is only 8 when the detection precision is improved, which is much higher than other approaches. Figure 4 shows the experimental results of our CCTSDB 2021 real-time detection network, which shows that our model is able to detect small traffic signs. Generally speaking, the proposed algorithm is highly accurate for object detection, and it can be used to find a good balance between the precision and the speed of recognition.

**Table 1:** Comprehensive test results of the CCTSDB 2021 (unit: %).

Method	P	R	MR	mAP	F1	FPS
SSD^21^	86.47	27.74	72.26	49.2	0.42	22.33
YOLOv3^18^	84.63	42.71	57.29	50.48	0.54	20.34
RetinaNet^38^	86.7	52.88	47.12	57.78	0.65	8.88
YOLOv4^19^	76.16	52.5	47.5	51.69	0.59	16.55
Faster R-CNN^14^	84.43	54.98	45.02	56.58	0.6	4.87
Dynamic R-CNN^39^	86.98	58.33	41.67	60.01	0.69	9.03
YOLOv5^20^	90.8	69.2	30.8	76.3	0.78	123.46
PSG-Yolov5^40^	91.6	80.9	24.7	80.2	0.82	114.35
Ours	91.9	78.6	21.4	82.8	0.84	115.37

In order to enrich the experimental results, we test our models in different classifications. Tables 2, 3 and 4 show the experimental results in various cases after dividing the test set, mAP is an average of the mean precision for all classes, so it is preferable to use a pair of measures in Tables 2, 3 and 4.

When the IoU threshold is 0.5, the results of P and R for each category measure are shown in Table 2. We find that our network is better than the baseline YOLOv5s in all indicators in CCTSDB 2021.

**Table 2:** Detection results of CCTSDB 2021 in different meaning categories (unit: %).

Method	Prohibitory		Warning		Mandatory	
	P	R	P	R	P	R
SSD^21^	80.75	24.84	86.15	26.6	92.5	31.79
YOLOv3^18^	88.15	42.31	82.37	54.39	83.37	31.44
RetinaNet^38^	93.68	52.46	81.96	63.66	84.47	42.53
YOLOv4^19^	75.85	50.11	76.2	59.4	76.42	47.99
Faster R-CNN^14^	90.6	55.51	83.63	67.93	79.05	41.49
Dynamic R-CNN^39^	95.44	57.53	84.86	70.55	80.65	46.91
YOLOv5^20^	90.9	69.8	90.4	82	91.1	55.8
PSG-Yolov5^40^	91.9	72.8	91.2	82.6	90.9	53.1
Ours	93.7	73.2	91.1	83.4	91.3	66.6

CCTSDB 2021 divided the size of traffic signs in the test set into five categories. The detection results of traffic signs of different sizes at an IoU threshold of 0.5 are shown in Table 3. Our model greatly improves the detection accuracy of XS and S size traffic signs, which are 12.5% and 3.8% higher than the original YOLOv5s, respectively. Therefore, our method can effectively improve the accuracy of small object detection.

**Table 3:** Detection results of CCTSDB 2021 in different object sizes (unit: %).

Method	XS		S		M		L		XL	
	P	R	P	R	P	R	P	R	P	R
SSD^21^	74.84	16.61	72.92	25.44	89.48	32.68	97.74	54.6	99.29	82.65
YOLOv3^18^	86.76	39.24	86.1	66.33	92.88	68.79	80.68	60.68	89.21	71.39
RetinaNet^38^	77.64	47	86.67	64.77	91.59	78.03	85.6	88.5	86.04	84.15
YOLOv4^19^	62.44	36.96	70.16	46.47	77.36	59.97	91.55	96.55	96.09	97.43
Faster R-CNN^14^	77.14	48.67	83.62	78.08	88.97	79.23	85.06	88.09	85.26	81.31
Dynamic R-CNN^39^	81.24	51.42	83.48	78.87	91.38	80.25	83.28	90.04	83.44	84.31
YOLOv5^20^	75.6	55.9	88.6	75.7	94.7	88.3	97.3	89	96.9	91.3
PSG-Yolov5^40^	79.8	67.3	90.4	78.6	95.9	88.5	97.9	90	97.2	90.4
Ours	88.1	79.8	92.4	81.4	96.2	88.6	98.3	90.7	97.2	89.6

In practical applications, weather conditions are complex, so the effectiveness of detection methods will vary with the weather environment in which the object is lo-cated. CCTSDB 2021 divided the weather conditions of the samples into six categories, and the detection results of the test set under six weather conditions with an IoU threshold of 0.5 are shown in Table 4. It can be seen that under sunny, snowy, and cloudy conditions, the P and R of the detection algorithm are relatively high, indicating that the algorithm is more effective in detecting without interference such as rain and fog. The experimental results indicate that under foggy weather conditions, the recog-nition accuracy and recall rate of road signal lights are relatively low. As shown in Figure 5, under some complex weather, our model can recognize the traffic signs not recognized by the original YOLOv5s, while improving the detection accuracy. In real life, weather conditions are complex and variable, and the performance of the detection algorithm varies with the weather environment in which the detected objects are located. CCTSDB 2021 divides the weather conditions of the samples into 6 categories, and the detection results of the test set under six weather conditions at an IoU threshold of 0.5 are shown in Table 4, we can see that the P and R of the detection algorithm are relatively high under sunny, snowy, and cloudy conditions, indicating that the algorithm is more effective in detecting without interference such as rain and fog. The precision and recall rate of the detection algorithm is relatively low in rain and fog and at night, indicating that rain and fog will have some influence on the detection of traffic signs, and also the visibility is relatively low at night, which is not conducive to the detection of traffic signs. However, the CA is introduced into our model, our model has high precision even in the case of blurred vision and certain occlusion. As shown in Fig. 5, under some complex weather, our model can recognize the traffic signs not recognized by the original YOLOv5s, while improving the detection accuracy.

**Table 4:** Detection results of CCTSDB 2021 in different weather conditions (unit: %).

Method	Sunny		Cloud		Night		Rain		Foggy		Snow	
	P	R	P	R	P	R	P	R	P	R	P	R
SSD^21^	90.56	32.65	84.45	21.77	85.22	24.59	57.88	27.53	85.42	32.99	95.65	28.1
YOLOv3^18^	92.01	64.03	87.12	44.65	75.98	34.81	91.17	31.55	88.66	56.39	87.54	70.59
RetinaNet^38^	90.71	75.37	93.43	53.92	81.09	43.81	67.98	39.55	69.45	64.86	90.18	88.49
YOLOv4^19^	83.83	53.95	74.24	52.92	67.65	32.47	22.43	13.41	85	37.43	64.32	40.84
Faster R-CNN^14^	85.47	77.42	92.74	57.61	76.89	47.87	61.35	34.61	77	67.09	96.27	91.12
Dynamic R-CNN^39^	86.26	78.92	93.87	58.4	83.7	52.26	64.21	41.13	70.57	69.52	96.25	89.48
YOLOv5^20^	95.9	85.1	94	81.2	86.1	60.6	47.9	46.7	64.8	81.3	96.1	80.7
PSG-Yolov5^40^	97.1	86.7	94.7	82.1	86.3	65.9	64.6	52.3	75.7	77.2	96.6	81.6
Ours	97.2	88.4	95.8	83.5	87.4	76.5	81.9	59	80.3	65.1	96.7	83.1

### Ablation study

To more intuitively demonstrate the better performance of the proposed method for traffic sign detection and recognition, we conduct the ablation study, and the results are shown in Table 5. Since we focus on the detection of small objects, in Table 5 we list the accuracy of the traffic signs for XS and S sizes.

**Table 5:** Overall performance on CCTSDB 2021 test (unit: %).

Method	Model	Param	FLOPs	FPS	Pxs	Ps	mAP
YOLOv5s	14.6M	7.193M	17.9G	123.46	75.6	88.6	76.3
YOLOv5s+CA	14.6M	7.193M	17.9G	123.46	80.9	89.7	78.6
YOLOv5s+sl(small object detection layer)	15.9M	8.039M	17.9G	115.37	85.4	91.2	80.9
Ours	15.9M	8.039M	17.9G	115.37	88.1	92.4	82.8

Table 5 shows the ablation result of incrementally adding the components training on the YOLOv5s model. As observed from the results, the standard YOLOv5s provides a detection mAP of 76.3%, Integrating the CA and the small object detection layer improves the mAP to 78.6% and 80.9%, respectively. The mAP of our method on the CCTSDB 2021 dataset is 6.5% higher than that of the YOLOv5s, which means the proposed method achieves impressive performance in object recognition. At the same time, the model size and parameters amount only slightly increase, and the FLOPs does not change, which means that the training speed of the improved network and the requirements for training equipment are unchanged. These ensure that our method can be easily deployed on vehicles and meet the requirements of real-time vehicle detection.

## Conclusion

In this paper, a lightweight traffic sign detection model is proposed, which is more balanced between detection speed and accuracy. It integrates the CA module in the backbone network of the YOLOv5s model and effectively uses the location information of the traffic sign to obtain the interesting area more accurately. For small traffic signs, we add a small object detection layer in the network to enhance the feature extraction ability of small objects and reduce the miss rate and false detection rate of small traffic signs. Finally, the original positioning losses were improved with Alpha-IoU, and the loss and gradient of high IoU objects are added by adjusting $\alpha$, thereby improving the bbox regression accuracy. The experimental results show that the method can achieve the most advanced performance at a faster reasoning speed, and the vehicle detection speed is 115 FPS. On the new test set of CCTSDB 2021, for small objects, the precision is 88.1%, and the recall rate is 79.8%, compared with the traditional YOLOv5s model, it is improved by 12.5% and 23.9% respectively. In various complicated weather conditions. The detection accuracy has also been greatly improved. However, in practical applications, unmanned driving has high requirements for object detection. In our works, the situation of occlusion was not considered. In the future, we plan to improve our framework through data augmentation and object persistence.

## Data Availability

The dataset of CCTSDB2021 used in this study are publicly available at https://github.com/csust7zhangjm/CCTSDB2021.

## Code availability

The source code will be made publicly available at https://github.com/yang-0706/ImprovedYOLOv5s.
